# Non-linear dose response effect of cathodal transcranial direct current stimulation on muscle strength in young healthy adults: a randomized controlled study

**DOI:** 10.1186/s13102-023-00621-7

**Published:** 2023-01-30

**Authors:** Oranich Vimolratana, Alexandra Lackmy-Vallee, Benchaporn Aneksan, Vimonwan Hiengkaew, Wanalee Klomjai

**Affiliations:** 1 Neuro Electrical Stimulation Laboratory (NeuE), Faculty of Physical Therapy, Mahidol University, 999 Phutthamonthon 4 Road, Nakhon Pathom, 73170 Thailand; 2 Faculty of Physical Therapy, Mahidol University, 999 Phutthamonthon 4 Road, Nakhon Pathom, 73170 Thailand; 3 Laboratoire d’Imagerie Biomédicale, LIB, CNRS, INSERM, Sorbonne Université, 75005 Paris, France

**Keywords:** Transcranial direct current stimulation, Cathodal transcranial direct current stimulation, Neuronal excitability, Muscle strength

## Abstract

**Background:**

Transcranial direct current stimulation (tDCS) is a technique that modulates brain excitability in humans. Increasing the stimulation intensity or duration within certain limits could enhance tDCS efficacy with a polarity-dependent effect; anodal stimulation increases cortical excitability, whereas cathodal stimulation decreases excitability. However, recent studies have reported a non-linear effect of cathodal tDCS on neuronal excitability in humans, and there is no conclusive result regarding the effect of cathodal tDCS on muscle performance.

**Methods:**

Our study aimed to investigate the immediate effects of different intensities (i.e., 1, 1.5, and 2 mA and sham tDCS) of cathodal tDCS on muscle strength in healthy participants. All participants [mean age 23.17 (3.90) years] were recruited and randomly allocated into four groups (1, 1.5, and 2 mA cathodal tDCS and sham tDCS). Muscle strength in bilateral upper and lower extremities was measured before and immediately after tDCS using a handheld dynamometer.

**Results:**

Our results showed that cathodal tDCS at 1 and 1.5 mA reduced muscle strength bilaterally in upper and lower extremity muscles, whereas stimulation at 2 mA tended to increase muscle strength on the dominant limb.

**Conclusion:**

These findings support the non-linear effects of cathodal tDCS on muscle strength, which should be considered for the clinical use of tDCS in motor rehabilitation.

*Trial registration*: NCT04672122, date of first registration 17/12/2020.

**Supplementary Information:**

The online version contains supplementary material available at 10.1186/s13102-023-00621-7.

## Background

Transcranial direct current stimulation (tDCS) is a noninvasive brain stimulation method that can modulate brain excitability in humans. The stimulation involves applying a weak direct current to the cortical area. The effects induced by tDCS are polarity dependent within a certain dose limit: anodal tDCS facilitates cortical neuron excitability, whereas cathodal tDCS decreases excitability [1–5]. Cathodal tDCS has been used for its inhibitory effect in clinical application. For example, it has been used to control hallucinations in patients with schizophrenia, a neuro-psychiatric disorder [6, 7]. In patients with attention-deficit hyperactivity disorder (ADHD), the cathode is applied over the left dorsolateral prefrontal cortex (DLPFC) to reduce high interhemispheric inhibition (IHI) towards the right hemisphere; thus resulting in enhance inhibitory control and attention [8, 9]. In stroke patients, the cathode is usually applied over the non-lesioned hemisphere to reduce over-excitability of the lesioned hemisphere and thus rebalance IHI [10, 11]. However, a meta-analysis reported an ineffectiveness of the unilateral montage of cathodal tDCS over the non-lesioned hemisphere in stroke [12], while other studies have indicated the usefulness of its inhibitory effect when used simultaneously with anodal stimulation over both hemispheres (bilateral montage) [13, 14]. The cathodal tDCS inhibitory effect has also been used for reducing involuntary movement in Tourette syndrome [15–17] and in individuals with encephalopathy [18], although one study reported no effect of cathodal tDCS in Tourette syndrome [19].

Regarding tDCS efficacy, the intensity of stimulation is one of the parameters that influences the effects of tDCS. However, a dose response for tDCS has not been fully established [20, 21]. Regarding the neurophysiological and motor responses, cathodal tDCS has shown conflicting results at different intensities. At low-intensity cathodal tDCS (1 mA), cortical excitability is reduced in humans [22, 23]; however, reversible effects have been observed (increased cortical excitability) at an intensity of 2 mA [22, 24–26]. However, some studies reported both reduction and enhancement of cortical excitability with 2 mA cathodal tDCS [27, 28]. While, 3 mA cathodal tDCS induced a reduction of cortical excitability [26]. Regarding the effect of cathodal tDCS on muscle performance in humans, previous studies reported a tendency to decrease muscle performance following cathodal tDCS at 1.5 mA and 2 mA [23, 24]. Another study showed slower performance during the early stimulation period (i.e., within the first 13 min of stimulation), and faster performance in the late stimulation period (i.e., after 13 min of stimulation) when 2 mA cathodal tDCS was applied for 20 min [25].

These conflicting results raise the question whether a common dose of cathodal tDCS used in clinic usually induces inhibitory effect. None have directly reported non-linearity of cathodal tDCS on clinical outcomes. We therefore explored the effect of cathodal tDCS on motor response by using the most common protocols (1–2 mA, 20 min). This study aimed to compare the immediate effects of 1, 1.5, and 2 mA cathodal tDCS applied to the primary motor cortex (M1) on muscle strength in healthy participants. We hypothesized a non-linear dose response effect of cathodal transcranial direct current stimulation on muscle strength, similar to the previously reported cortical response; tDCS 1 and 1.5 mA cathodal tDCS would decrease muscle strength, whereas 2 mA cathodal tDCS would increase strength. The results of this study could serve as evidence regarding stimulation effect of cathodal tDCS on the clinical outcome measured as muscle strength and provide an awareness regarding intensity selection in cathodal tDCS for future research.

## Methods

### Participants

**Fig. 1:**
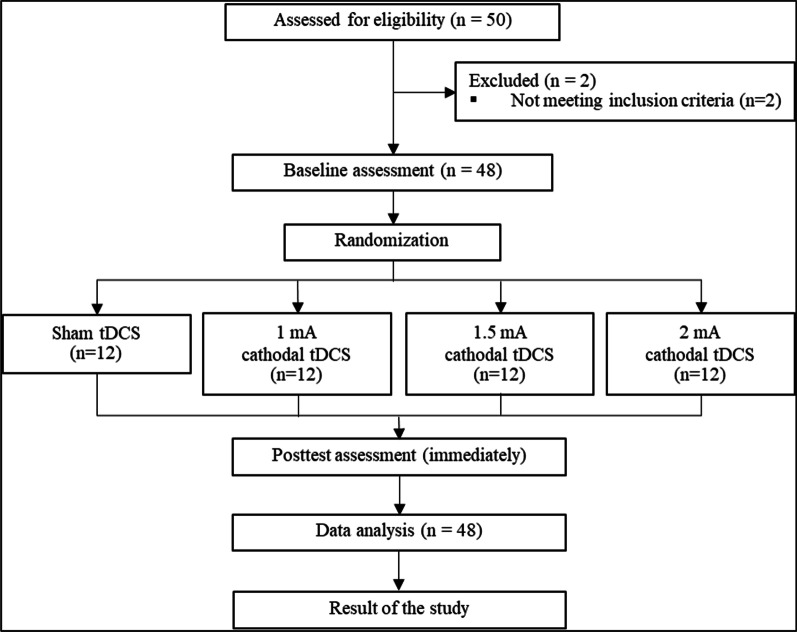
Flowchart of the study procedure

Forty-eight healthy adults aged 18–40 years participated in this study. Participants were recruited and randomly split into four groups (sham, 1 mA, 1.5 mA, and 2 mA cathodal tDCS). This study was a randomized controlled trial. A randomization was performed by an independent researcher who was not involved in tDCS application and outcome measurement. Closed-opaqued envelopes with number of groups were used for randomization. All participants and an assessor were blinded to the groups. In each participant, muscle strength (i.e., elbow flexor, elbow extensor, wrist extensor, hip flexor, knee flexor, knee extensor, ankle dorsiflexor, and ankle plantar flexor muscles) was measured in both the dominant and non-dominant upper and lower extremities before and after the intervention (see Fig. 1). The inclusion criteria were as follows: healthy adults aged 18–40 years, right-hand dominant (screened by the Edinburg Handedness Inventory), and no injury to either limb for the past 6 months. Exclusion criteria were as follows: metal implantation, intracranial shunt, cochlear implant or cardiac pacemaker, an open wound or infectious wound around the scalp, history of neurological symptoms (i.e., seizures, weakness, loss of sensation, or unclear history of past illness), pain in the muscle groups being evaluated, and history of surgery in the limbs being evaluated. All participants were instructed to avoid caffeine consumption within 24 h prior to the study. If the participant consumed caffeine prior to the intervention, they were rescheduled for another day. All participants were non-smokers.

This study was approved by the Mahidol University Central Institutional Review Board (MU-CIRB 2020/314.0210) and was registered on ClinicalTrials.gov (ID NCT04672122, date of first registration 17/12/2020). The work described has been carried out in accordance with The Code of Ethics of the World Medical Association (Declaration of Helsinki) for experiments involving humans. All participants signed written informed consent for participation in the study and publication of the results. Data was collected at Faculty of Physical Therapy, Mahidol University.

### Muscle strength measurement

**Fig. 2:**
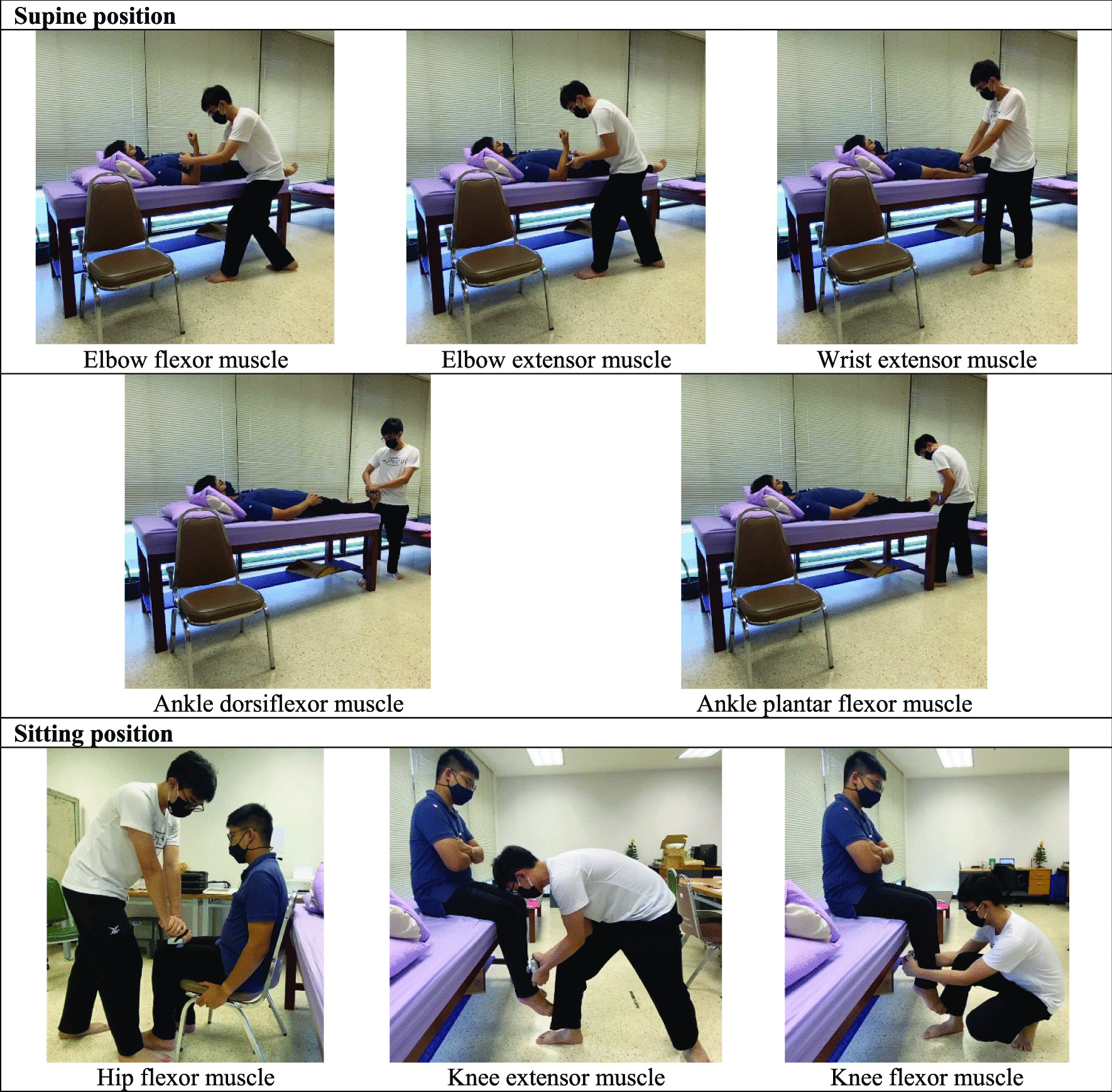
Positions used to evaluate muscle strength of upper and lower extremities. Muscles strength were evaluated in newton (N). In supine position, we evaluated elbow flexor, elbow extensor, wrist extensor, ankle dorsiflexor and ankle plantar flexor muscles. Participant lied in supine position with arm beside their trunk, elbow flexed at 90°, and wrist in neutral position. Dynamometer were placed on anterior forearm, proximal to wrist joint, to assess elbow flexor and on the posterior forearm to assess elbow extensor. For wrist extensor muscle, participants remained in supine position with arm beside trunk, elbow extend with forearm in pronation and wrist in neutral position. Dynamometer were placed at posterior aspect of hand, proximal to metacarpophalangeal joints. For ankle dorsiflexor and plantar flexor muscles, participants were instructed to lied in supine position with hip and knee full extension and ankle in neutral position. Dynamometer were placed at posterior or anterior aspect of foot, proximal to metatarsophalangeal joints to assess ankle dorsiflexor and ankle plantar flexor muscles, respectively. In sitting position, we evaluated hip flexor, knee extensor, and knee flexor muscles. Participants were instructed to sit with hip and knee flexion at 90°. Dynamometer was placed over anterior thigh, proximal to knee joint, to evaluate hip flexor muscle. For knee flexor and extensor muscles, participants were instructed to remained in sitting position. Dynamometer was placed over the anterior or posterior aspect of leg, proximal to the ankle joint, to evaluate knee extensor and knee flexor muscles, respectively

A Lafayette handheld-dynamometer model 01165 (Lafayette Instrument Company, Lafayette, IN, USA) was used to evaluate bilateral upper and lower extremity muscle strength. Participants were positioned in two different positions to assess strength: supine and sitting. The testing positions were selected to minimize the effect of gravity and the compensation of each muscle during measurement [29, 30]. The supine position was selected for elbow flexor, elbow extensor, wrist extensor, ankle dorsiflexor, and ankle plantar flexor muscle strength measurement. The sitting position was selected for hip flexor, knee flexor, and knee extensor muscle strength measurement. All testing positions are shown in Fig. 2. The participants were permitted to rest for at least 1 min or until they recovered from fatigue after each measurement. The positions used to evaluate each muscle are also shown in Fig. 1. Each muscle was evaluated twice, and the best trial was used for statistical analysis. Muscle strength was measured before and after tDCS. Measurement was started within 1 min after tDCS ended; the total measurement procedure lasted approximately 20 min, as cortical excitability can last for up to 30 min after cessation of cathodal stimulation [25, 26].

### Transcranial direct current stimulation

**Fig. 3:**
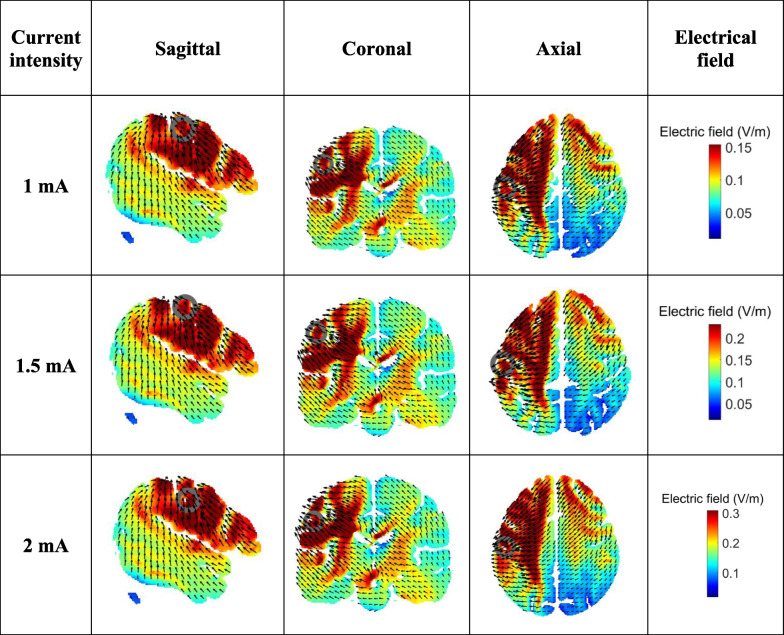
Electrical fields of cathodal transcranial direct current stimulation (tDCS) in each group. A target electrode (cathodal) placed over the left motor cortex (C3) and a reference electrode (anodal) placed over the right supraorbital area (Fp2). Finite element models of tDCS application. Slice positions (ROAST’s MNI152 head) are taken at MNI coordinates of − 53, − 16, and 47 for sagittal, coronal, and axial slices, respectively. In the sagittal and coronal views, the gray circle represents the position of the electrode, and the black arrows represent electrical current flow in the brain. The red color represents the higher electric field, and the blue color represents the lower electric field in the brain. Compared to 1.5 and 2 mA cathodal tDCS, in 1 mA (coronal view), the electric field is higher

Cathodal transcranial direct current stimulation (MINDD STIM; Ybrain Inc., Korea) was used in this study. The cathode was applied over the dominant primary motor cortex (M1) at C3, and the anode was applied over the contralateral orbital area (Fp2) following: 10–20 EEG electrode placement. The 35 cm^2^ saline-soaked electrodes were used with intensities of 1, 1.5, and 2 mA in each group. Each group received stimulation for 20 min with an active beeping sound throughout the stimulation. Sham mode was selected for the sham group. The electrical stimulation was applied only for the first 30 s and was automatically switched off and remained in position for 20 min with an active beeping sound for sham stimulation. The realistic volumetric approach to simulate transcranial electric stimulation (ROAST) pipeline [31] was used to demonstrate the tDCS-induced electrical field in this study (Fig. 3). Information regarding adverse effects perceived during and after stimulation (such as tingling, itching, burning sensation, headache, redness, drowsiness, etc.) was obtained at the end of each session using a questionnaire suggested by Brunoni and colleagues [32].

### Sample size calculation

Sample size was calculated based on the determination of input parameters for a four-group comparison (effect size f = 0.5, α error probability *p* = 0.05, power = 0.8), based on one-way analysis of variance (ANOVA) analysis. The determined effect size was the intermediate effect size for statistically significant results reported in tDCS studies [33]. The sample size was calculated to be 12 participants per group. Therefore, we recruited 48 participants.

### Statistical analyses

The comparison of baseline characteristics between groups was analyzed using the Chi-square test and Kruskal–Wallis test. The Shapiro–Wilk test was used to check the normality of the data. Muscle strength data were expressed as a percentage of the baseline value to estimate the changes before statistical analysis. The formula used to calculate the percentage change was ([posttest − pretest)/pretest] × 100). Comparisons within groups (pretest vs. posttest) were performed using the Wilcoxon signed-rank test, and comparisons between groups (sham vs. 1 mA vs. 1.5 mA, and 2 mA) were performed using the Kruskal–Wallis test (post hoc analysis by Tukey’s test). Statistical significance was set at *p* < 0.05. Characteristic data are presented as median (IQR), and outcome measurement data are presented as mean (SD, standard deviation). The effect sizes for within group were calculated from the formula *r* = *Z*/$\sqrt{n}$). The values 0.1, 0.25, and 0.40 were interpreted as small, medium, and large effect, respectively [34, 35].

## Results

Forty-eight healthy participants underwent cathodal tDCS protocols. There were no significant differences between the groups in baseline characteristics except age in the 1 mA cathodal tDCS group (Table 1). Mild adverse effects observed included tingling sensation (active 73.33%, sham 0%), skin redness (active 46.67%, sham 0%), sleepiness (active 23.33%, sham 10%), burning sensation (active 16.67%, sham 0%), and itching (active 13.33%, sham 0%) (See Additional file 1).

**Table 1:** Participants characteristics [Median (IQR)]

Variables	1 mA cathodal tDCS	1.5 mA cathodal tDCS	2 mA cathodal tDCS	Sham tDCS	*p* value
Number of participants	12	12	12	12	–
Sex (male:female)	2:10	0:12	0:12	2:10	0.225^a^
Age (years)	26.50 (25.00, 29.75)	21.00 (20.25, 21.75)	21.00 (20.25, 21.00)	22.00 (19.50, 24.00)	< 0.001^b^
Weight (kg)	54.15 (47.00, 60.50)	53.00 (47.75, 58.00)	52.00 (45.50, 58.75)	52.00 (45.00, 68.00)	0.937^b^
Height (m)	1.61 (1.57, 1.65)	1.59 (1.56, 1.63)	1.60 (1.58, 1.69)	1.61 (1.57, 1.68)	0.852^b^
BMI	21.81 (18.61, 22.76)	19.98 (19.17, 22.04)	19.59 (18.52, 20.35)	21.10 (17.02, 24.66)	0.598^b^

^a^Testing by using Chi square test

^b^Testing by using Kruskal–Wallis test

### Effect of cathodal tDCS on the dominant limbs

#### Dominant upper extremities muscles

**Fig. 4:**
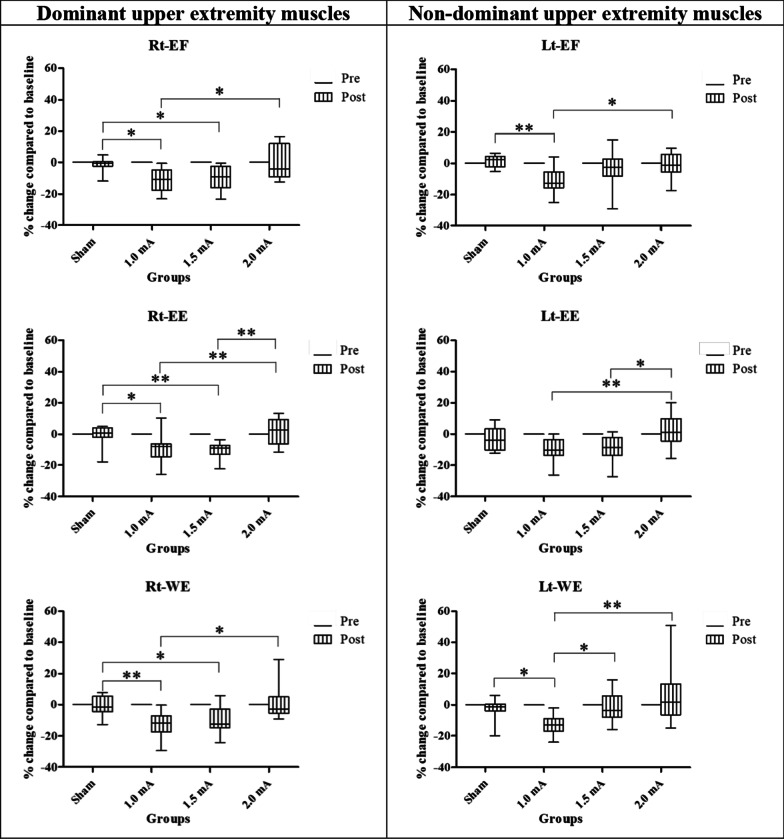
Box-and-whisker plots of upper extremities muscle strength showing the percentage change from baseline for four groups at pretest and posttest. The box’s width represents the interquartile range, the horizontal line within the boxes represents the median, the upper and below whiskers represent the minimum and maximum values. (*and **represent significant difference between groups at < 0.05 and < 0.01, respectively)

The Wilcoxon signed-rank test revealed a significant strength reduction of all the muscles for both 1 and 1.5 mA cathodal tDCS. No significant differences were found in sham and 2 mA cathodal tDCS groups (Table 2). The Kruskal–Wallis test showed significant differences between groups at posttest for all muscles [H(3) = 14.378 (*p* = 0.002), H(3) = 18.667 (*p* < 0.001), H(3) = 16.850 (*p* < 0.001) for elbow flexor, elbow extensor, and wrist extensor muscles, respectively] (Fig. 4; Table 2). Compared to the pretest, in the posttest, most effect sizes were medium to large (data are presented in Table 2). These results suggest an immediate effect of 1 and 1.5 mA cathodal tDCS on the reduction of muscle strength of dominant upper extremity muscles.

**Table 2:** Percentage changes from baseline of the upper extremity muscle strength on the dominant limb are presented as mean (SD) at posttest (POST) in each group

Muscles of the dominant limb	Groups	Percentage change from baseline	Within group comparison			Between group comparison		
		POST [Mean (SD)]	Z	*p* value	Effect size	Overall	Post hoc	
							Comparison groups	*p* value
Elbow flexor	Sham tDCS	− 0.36 (3.04)	− 1.334	0.204	0.39	0.002**	Sham versus 1 mA	0.013*
	1.0 mA ctDCS	− 9.94 (7.27)	− 3.059	< 0.001***	0.88		Sham versus 1.5 mA	0.034*
	1.5 mA ctDCS	− 9.42 (7.11)	− 3.059	< 0.001***	0.88		Sham versus 2 mA	0.972
	2.0 mA ctDCS	0.15 (10.52)	0.392	0.733	0.11		1 mA versus 1.5 mA	0.989
							1 mA versus 2 mA	0.047*
							1.5 mA versus 2 mA	0.104
Elbow extensor	Sham tDCS	1.21 (3.55)	0.471	0.677	0.14	< 0.001***	Sham versus 1 mA	0.037*
	1.0 mA ctDCS	− 10.17 (10.56)	− 2.432	0.012*	0.70		Sham versus 1.5 mA	0.009**
	1.5 mA ctDCS	− 10.75 (5.44)	− 3.059	< 0.001***	0.88		Sham versus 2 mA	0.996
	2.0 mA ctDCS	2.96 (7.59)	0.706	0.519	0.20		1 mA versus 1.5 mA	0.966
							1 mA versus 2 mA	0.019*
							1.5 mA versus 2 mA	0.004**
Wrist extensor	Sham tDCS	0.62 (5.14)	− 0.471	0.677	0.14	< 0.001***	Sham versus 1 mA	0.006**
	1.0 mA ctDCS	− 11.49 (8.03)	− 3.059	< 0.001***	0.88		Sham versus 1.5 mA	0.048*
	1.5 mA ctDCS	− 8.79 (9.44)	− 2.432	0.012*	0.70		Sham versus 2 mA	0.999
	2.0 mA ctDCS	− 0.92 (7.01)	− 0.196	0.850	0.06		1 mA versus 1.5 mA	0.897
							1 mA versus 2 mA	0.010*
							1.5 mA versus 2 mA	0.072

*Indicated *p* < 0.05, ** indicated *p* < 0.01, and *** indicated *p* < 0.001

#### Dominant lower extremities muscles

**Fig. 5:**
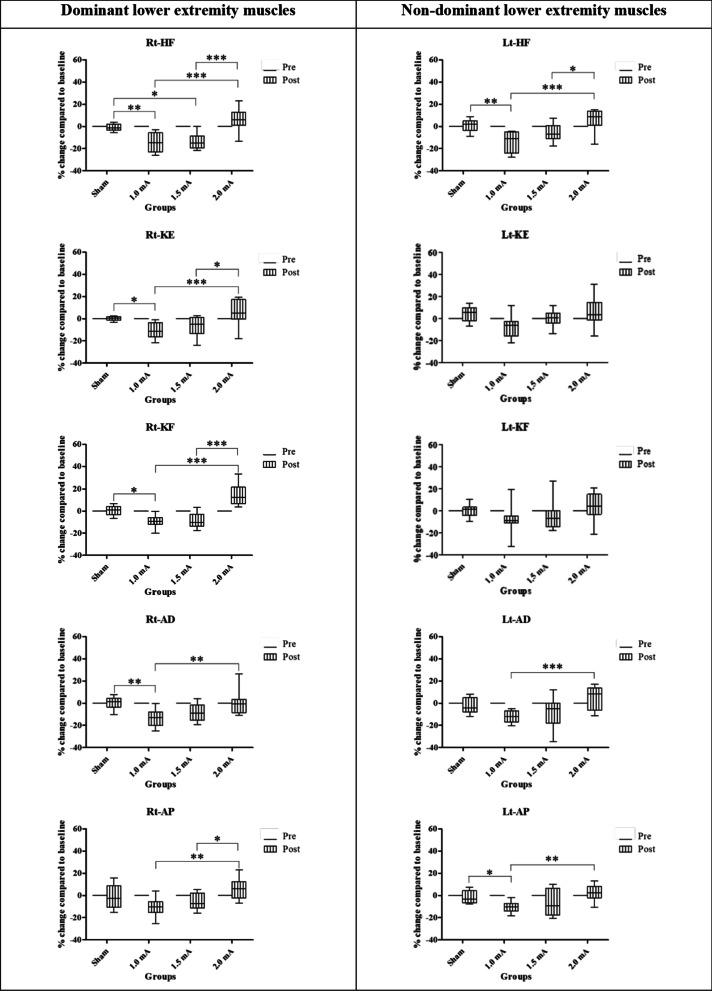
Box-and-whisker plots of lower extremities muscle strength showing the percentage change from baseline for four groups at pretest and posttest. The box’s width represents the interquartile range, the horizontal line within the boxes represents the median, the upper and below whiskers represent the minimum and maximum values. (*, ** and ***represent significant difference between groups at < 0.05, < 0.01 and < 0.001, respectively)

The Wilcoxon signed-rank test revealed a significant strength reduction of all the muscles for both 1 and 1.5 mA cathodal tDCS. There were no significant differences in sham and 2 mA cathodal tDCS for most muscles except an enhancement of knee flexor muscle strength in 2 mA cathodal tDCS group (*p* < 0.001) (Table 3). The Kruskal–Wallis test showed significant differences between groups at posttest for all muscles [H(3) = 29.407 (*p* < 0.001), H(3) = 19.194 (*p* < 0.001), H(3) = 34.077 (*p* < 0.001), H(3) = 17.522 (*p* < 0.001), H(3) = 14.792 (*p* = 0.002) for hip flexor, knee flexor, knee extensor, ankle dorsiflexor, and ankle plantar flexor muscles, respectively] (Fig. 5; Table 3). Most effect sizes were larger for the posttest than for the pretest (data are presented in Table 3). These results showed immediate effect of 1 and 1.5 mA cathodal tDCS on the reduction of muscle strength, while 2 mA cathodal tDCS enhanced knee flexor muscle strength on dominant lower extremities muscles.

**Table 3:** Percentage changes from baseline of the lower extremity muscle strength on the dominant limb are presented as mean (SD) at posttest (POST) in each group

Muscles of the dominant limb	Groups	Percentage change from baseline	Within group comparison			Between group comparison		
		POST [Mean (SD)]	Z	*p* value	Effect size	Overall	Post hoc	
							Comparison groups	*p* value
Hip flexor	Sham tDCS	0.02 (2.78)	− 0.746	0.470	0.22	< 0.001***	Sham versus 1 mA	0.007**
	1.0 mA ctDCS	− 13.01 (8.74)	− 3.059	< 0.001***	0.88		Sham versus 1.5 mA	0.018*
	1.5 mA ctDCS	− 12.45 (7.24)	− 2.981	< 0.001***	0.86		Sham versus 2 mA	0.564
	2.0 mA ctDCS	9.46 (7.45)	1.961	0.052	0.57		1 mA versus 1.5 mA	0.993
							1 mA versus 2 mA	< 0.001***
							1.5 mA versus 2 mA	< 0.001***
Knee extensor	Sham tDCS	− 0.17 (2.01)	0.392	0.733	0.11	< 0.001***	Sham versus 1 mA	0.018*
	1.0 mA ctDCS	− 8.45 (5.93)	− 3.059	< 0.001***	0.88		Sham versus 1.5 mA	0.702
	1.5 mA ctDCS	− 5.39 (8.46)	− 2.275	0.021*	0.66		Sham versus 2 mA	0.648
	2.0 mA ctDCS	7.27 (10.68)	1.647	0.110	0.48		1 mA versus 1.5 mA	0.648
							1 mA versus 2 mA	< 0.001***
							1.5 mA versus 2 mA	0.023*
Knee flexor	Sham tDCS	1.21 (3.90)	0.314	0.791	0.09	< 0.001***	Sham versus 1 mA	0.037*
	1.0 mA ctDCS	− 8.74 (5.33)	− 3.059	< 0.001***	0.88		Sham versus 1.5 mA	0.068
	1.5 mA ctDCS	− 7.82 (7.26)	− 2.747	0.003**	0.79		Sham versus 2 mA	0.079
	2.0 mA ctDCS	15.50 (9.39)	3.059	< 0.001**	0.88		1 mA versus 1.5 mA	0.996
							1 mA versus 2 mA	< 0.001***
							1.5 mA versus 2 mA	< 0.001***
Ankle dorsiflexor	Sham tDCS	− 0.25 (6.25)	0.157	0.910	0.05	< 0.001***	Sham versus 1 mA	0.002**
	1.0 mA ctDCS	− 14.97 (6.36)	− 3.059	< 0.001***	0.88		Sham versus 1.5 mA	0.071
	1.5 mA ctDCS	− 8.43 (8.97)	− 2.510	0.009**	0.73		Sham versus 2 mA	0.990
	2.0 mA ctDCS	− 0.67 (7.05)	− 0.078	0.970	0.02		1 mA versus 1.5 mA	0.676
							1 mA versus 2 mA	0.006**
							1.5 mA versus 2 mA	0.144
Ankle plantarflexor	Sham tDCS	− 2.51 (10.23)	− 0.314	0.791	0.09	0.002**	Sham versus 1 mA	0.119
	1.0 mA ctDCS	− 8.38 (6.61)	− 2.903	0.001**	0.84		Sham versus 1.5 mA	0.657
	1.5 mA ctDCS	− 5.07 (7.98)	− 2.197	0.027*	0.63		Sham versus 2 mA	0.472
	2.0 mA ctDCS	6.63 (10.01)	1.647	0.110	0.48		1 mA versus 1.5 mA	0.711
							1 mA versus 2 mA	0.001**
							1.5 mA versus 2 mA	0.047*

*Indicated *p* < 0.05, **indicated *p* < 0.01, and *** indicated *p* < 0.001

### Effect of cathodal tDCS on the non-dominant limbs

#### The non-dominant upper extremities muscles

The Wilcoxon signed-rank test revealed a significant strength reduction of all the muscles in the 1 mA cathodal tDCS group. While 1.5 mA cathodal tDCS groups only significantly reduced the strength of the elbow extensor muscle (*p* = 0.002). There was no significant reduction in the sham and 2 mA cathodal tDCS group (Table 4). The Kruskal–Wallis test showed significant difference between groups at posttest [H(3) = 14.309 (*p* = 0.003), H(3) = 12.642 (*p* = 0.005), H(3) = 15.704 (*p* = 0.001) for elbow flexor, elbow extensor, and wrist extensor muscles, respectively] (Fig. 4; Table 4). Compared to the pretest, in the posttest, most effect sizes were medium to large (data are presented in Table 4). These results suggest that 1 mA cathodal tDCS also reduced muscle strength of the non-stimulated muscles.

**Table 4:** Percentage changes from baseline of the upper extremity muscle strength on the non-dominant limb are presented as mean (SD) at posttest (POST) in each group

Muscles of the non-dominant limb	Groups	Percentage change from baseline	Within group comparison			Between group comparison		
		POST [Mean (SD)]	Z	*p* value	Effect size	Overall	Post hoc	
							Comparison groups	*p* value
Elbow flexor	Sham tDCS	1.04 (3.98)	0.863	0.424	0.25	0.003**	Sham versus 1 mA	0.001**
	1.0 mA ctDCS	− 11.35 (8.25)	− 2.903	0.001**	0.84		Sham versus 1.5 mA	0.360
	1.5 mA ctDCS	− 4.29 (12.42)	− 1.255	0.233	0.36		Sham versus 2 mA	0.746
	2.0 mA ctDCS	− 0.33 (6.02)	− 0.392	0.733	0.11		1 mA versus 1.5 mA	0.178
							1 mA versus 2 mA	0.040*
							1.5 mA versus 2 mA	0.923
Elbow extensor	Sham tDCS	− 1.58 (6.98)	− 1.569	0.129	0.45	0.005**	Sham versus 1 mA	0.305
	1.0 mA ctDCS	− 10.84 (8.91)	− 2.934	< 0.001***	0.85		Sham versus 1.5 mA	0.454
	1.5 mA ctDCS	− 7.86 (8.08)	− 2.824	0.002**	0.82		Sham versus 2 mA	0.499
	2.0 mA ctDCS	4.49 (9.32)	0.863	0.424	0.25		1 mA versus 1.5 mA	0.994
							1 mA versus 2 mA	0.009**
							1.5 mA versus 2 mA	0.021*
Wrist extensor	Sham tDCS	− 1.11 (3.87)	− 1.412	0.176	0.41	0.001**	Sham versus 1 mA	0.020**
	1.0 mA ctDCS	− 13.47 (5.95)	− 3.059	< 0.001***	0.88		Sham versus 1.5 mA	0.998
	1.5 mA ctDCS	− 0.83 (9.47)	− 0.784	0.470	0.23		Sham versus 2 mA	0.833
	2.0 mA ctDCS	− 0.33 (9.19)	0.784	0.470	0.23		1 mA versus 1.5 mA	0.034*
							1 mA versus 2 mA	0.001***
							1.5 mA versus 2 mA	0.737

*Indicated *p* < 0.05, **indicated *p* < 0.01, and ***indicated *p* < 0.001

#### Non-dominant lower extremities muscles

The Wilcoxon signed-rank test revealed a significant strength reduction of all the muscles after 1 mA cathodal tDCS. While 1.5 mA cathodal tDCS only significantly reduced hip flexor muscle strength. There was no significant reduction in sham and 2 mA cathodal tDCS (Table 5). The Kruskal–Wallis test showed significant differences between groups at posttest [H(3) = 22.844 (*p* < 0.001), H(3) = 10.893 (*p* = 0.012), H(3) = 12.859 (*p* = 0.005), H(3) = 15.986 (*p* = 0.001), H(3) = 14.291 (*p* = 0.003) for hip flexor, knee extensor, knee flexor, ankle dorsiflexor, and ankle plantar flexor muscles, respectively] (Fig. 4; Table 5). Compared to the pretest, in the posttest, most effect sizes were medium to large (data are presented in Table 5). These results suggest that 1 mA cathodal tDCS also reduced muscle strength of the non-stimulated lower extremities muscles.

**Table 5:** Percentage changes from baseline of the lower extremity muscle strength on the non-dominant limb are presented as mean (SD) at posttest (POST) in each group

Muscles of the non-dominant limb	Groups	Percentage change from baseline	Within group comparison			Between group comparison		
		POST [Mean (SD)]	Z	*p* value	Effect size	Overall	Post hoc	
							Comparison groups	*p* value
Hip flexor	Sham tDCS	1.09 (5.62)	0.942	0.380	0.27	< 0.001***	Sham versus 1 mA	0.008**
	1.0 mA ctDCS	− 11.38 (8.15)	− 3.059	< 0.001***	0.88		Sham versus 1.5 mA	0.263
	1.5 mA ctDCS	− 4.40 (7.19)	− 2.118	0.034*	0.61		Sham versus 2 mA	0.592
	2.0 mA ctDCS	9.98 (5.04)	1.883	0.064	0.54		1 mA versus 1.5 mA	0.536
							1 mA versus 2 mA	< 0.001***
							1.5 mA versus 2 mA	0.011*
Knee extensor	Sham tDCS	4.66 (6.60)	1.883	0.064	0.54	0.012*	Sham versus 1 mA	0.024*
	1.0 mA ctDCS	− 4.79 (8.99)	− 2.118	0.034*	0.61		Sham versus 1.5 mA	0.676
	1.5 mA ctDCS	1.10 (6.58)	0.235	0.850	0.07		Sham versus 2 mA	1.000
	2.0 mA ctDCS	7.06 (10.63)	1.490	0.151	0.43		1 mA versus 1.5 mA	0.321
							1 mA versus 2 mA	0.021**
							1.5 mA versus 2 mA	0.648
Knee flexor	Sham tDCS	− 0.20 (5.81)	0.000	1.000	0.00	0.005**	Sham versus 1 mA	0.066
	1.0 mA ctDCS	− 6.06 (10.33)	− 2.275	0.021*	0.66		Sham versus 1.5 mA	0.243
	1.5 mA ctDCS	− 3.95 (12.64)	− 1.804	0.077	0.52		Sham versus 2 mA	0.933
	2.0 mA ctDCS	7.19 (9.39)	1.255	0.233	0.36		1 mA versus 1.5 mA	0.933
							1 mA versus 2 mA	0.012*
							1.5 mA versus 2 mA	0.097
Ankle dorsiflexor	Sham tDCS	− 1.72 (7.40)	− 1.177	0.266	0.34	0.001**	Sham versus 1 mA	0.052
	1.0 mA ctDCS	− 11.80 (5.78)	− 3.059	< 0.001***	0.88		Sham versus 1.5 mA	0.758
	1.5 mA ctDCS	− 6.71 (13.29)	− 1.961	0.052	0.57		Sham versus 2 mA	0.550
	2.0 mA ctDCS	5.27 (9.28)	1.647	0.110	0.48		1 mA versus 1.5 mA	0.397
							1 mA versus 2 mA	< 0.001***
							1.5 mA versus 2 mA	0.097
Ankle plantarflexor	Sham tDCS	− 1.74 (5.75)	− 0.863	0.424	0.25	0.003**	Sham versus 1 mA	0.040*
	1.0 mA ctDCS	− 10.01 (3.91)	− 3.059	< 0.001***	0.88		Sham versus 1.5 mA	0.352
	1.5 mA ctDCS	− 7.32 (12.21)	− 1.804	0.077	0.52		Sham versus 2 mA	0.879
	2.0 mA ctDCS	2.68 (6.80)	1.255	0.233	0.36		1 mA versus 1.5 mA	0.746
							1 mA versus 2 mA	0.004**
							1.5 mA versus 2 mA	0.079

*Indicated *p* < 0.05, **indicated *p* < 0.01, and ***indicated *p* < 0.001

## Discussion

Our results demonstrated that different cathode intensities applied over the dominant hemisphere induced different changes in muscle strength in healthy participants. Cathodal tDCS at 1 mA reduced the strength of all muscles in the dominant and the non-dominant limbs. Stimulation with 1.5 mA cathodal tDCS reduced all upper and lower extremities muscle strength on the dominant (contralateral) limbs, and the strength of the elbow extensor of the non-dominant (ipsilateral) limbs. Meanwhile, 2 mA of cathodal tDCS resulted in an increase of muscle strength in the knee flexor muscles on the dominant extremity.

Cathodal stimulation was placed over the M1 of the dominant hemisphere (C3) where is more related to the upper limb area. However, from the computational electrical field (Fig. 3), tDCS can reach a wide area including the lower limb M1 for all tested intensities. This outcome is probably caused by a non-focal effect of tDCS. This finding is consistent with our previous studies in patients with stroke showing that anodal, cathodal, and dual tDCS over the primary motor cortex in the upper extremity (C3 or C4) could induce motor performance change in both upper and lower limbs [14, 36].

For 1 mA cathodal tDCS, we found a significant reduction of muscle strength in both the dominant and non-dominant extremities of righted-handed people. A previous study with right-handed participants showed that 1 mA cathodal tDCS over the dominant and the non-dominant hemispheres could decrease cortical excitability by ~ 20% in the stimulated hemisphere [37]. Moreover, cathodal tDCS at low intensity (1 mA) for 20 min led to a decrease in cortical excitability in the stimulated motor area, which lasted for 30 min after stimulation [22, 26]. These results are in line with ours, which showed a reduction in muscle strength of the dominant limb after 1 mA cathodal tDCS.

For 1.5 mA cathodal tDCS, our results showed a significant strength reduction of all the dominant upper and lower extremities muscles. These findings are consistent with a previous study that reported a decrease in cortical excitability after 1.5 mA cathodal tDCS over the M1 [5]. A previous study with healthy participants also reported a tendency of decreased muscle performance of the upper extremity in the tDCS group following 1.5 mA cathodal stimulation for 10 min [38]. In addition, a previous study in volleyball players reported decreasing of motor learning following 1.5 mA cathodal stimulation over the dorsolateral prefrontal cortex for 10 min prior to training [39].

In the 2 mA cathodal tDCS group, we found a significant increase in the knee flexor muscle and a tendency of increased muscle strength for most muscles in the dominant limb. Previous studies have reported muscle strength differences measured by cortical excitability following 2 mA cathodal tDCS over M1 [22, 26]. For instance, two studies reported a reverse effect of 20 min of 2 mA cathodal tDCS on cortical excitability in healthy adults [22, 26], while another study reported a tendency of decreased lower extremity muscle strength after 10 min of cathodal tDCS at 2 mA over M1 [40]. The underlying mechanism for the reversal effect of cathodal tDCS might be related to calcium levels. A recent study suggested that calcium channel dynamics are involved in the non-linear after-effect of high intensity cathodal tDCS (3 mA) [41]. Plasticity of tDCS involved with the activation of *N*-methyl-*D*-aspartate (NMDA) receptors and post-synaptic calcium concentration in the brain [41, 42]. Low intracellular calcium concentration leads to long-term depression (LTD) in neurons. While high intracellular calcium concentration leads to long-term potentiation (LTP). In addition, there are also “no man’s lands” zones in which calcium concentration overflows the LTD and LTP zones and does not result in plasticity [42, 43]. The conversion effect of 2 mA cathodal tDCS might result from the calcium concentration overflow to the LTP zone, not the LTD zone. Different intensities of cathodal tDCS (1, 1.5, and 2 mA) might result in different calcium concentrations at the post-synapsis and resulted in different effects of cathodal tDCS. This hypothesis should be explored in the future.

We also found changes in upper limb muscle strength (i.e., elbow flexor, elbow extensor, and wrist extensor) of the non-dominant extremities following 1 and 1.5 mA cathodal tDCS. Several possible explanations are described below. One explanation could be the non-focality effect of tDCS. In the present study, a large electrode size (35 cm^2^) was used for both the active and reference electrodes, which might have resulted in widespread cortical excitability changes. A large active electrode size could lead to a non-focality effect of tDCS [44, 45]. It was reported that cathodal tDCS induced changes in cortical excitability in both stimulated and non-stimulated hemispheres [46], and induced change in the alpha band power, which is related to the preparation of movement [47] in both stimulated and non-stimulated hemispheres [48]. A second possible explanation is related to the motor control of the nervous system. Corticospinal actions in upper limb motoneurons normally receive commands from the contralateral hemisphere. The reticulospinal tract is also in a position to influence motoneurons projecting to both the proximal and distal upper limb muscles [49], which receive the signal from both dominant and non-dominant hemispheres [50]. The signal is projected towards the ipsilateral cervical propriospinal system in the spinal cord [51]. These actions might have played a role in the change in muscle strength of the non-dominant (ipsilateral) limb observed in the present study. Previous studies have also reported that cathodal tDCS over M1 (1 mA for 20 min) induced excitability changes in ipsilateral propriospinal premotor neurons in healthy participants [50], and enhanced ipsilateral muscle selection for specific tasks in healthy participants [52].

Previous studies by Batsikadze et al. and Mosayebi Samani et al. reported non-linear neurophysiological changes after cathodal tDCS at different intensities [22, 26]. Here, muscle strength, our clinical outcome, showed similar changes. These findings emphasize a collateral response of both neurophysiological and clinical outcomes following cathodal tDCS in healthy populations. This study provides evidence that sheds light on the importance of the selection of the appropriate intensity of cathodal tDCS in clinical practice. For instance, clinicians should consider whether cathodal tDCS or dual stimulation (anodal and cathodal applied simultaneously) at 2 mA is appropriate to use to rebalance IHI post-stroke, or to reduce involuntary movement in Huntington’s chorea, and chronic tic disorders like Tourette syndrome since it may induce reverse effects. Further studies that include other populations such as neurological patients are necessary for a complete perspective.

### Limitations of the study

There are limitations to our study that should be considered. This study was performed on healthy young participants. Therefore, it is unlikely that the results can be generalized to other populations. Most recruited participants were female, which may also have affected the tDCS results. A fluctuation of hormones and neurotransmitters induced by a menstrual cycle affects the cortical excitability in the human brain [53]. There was significant difference of age in 1 mA ctDCS group compared to other groups in this study which may affect cathodal tDCS effect as age has been noted as one of variable that can affect tDCS response [54]. Matched pair for age is suggested for a future study. Here, there was no blinding assessment of participants. With higher intensity of stimulation, there is a higher probability of correctly identifying the group allocation, especially using a within-subject design [55, 56]. However, our current study uses a between-subject design. In addition, recent study by Stankovic et al. reported that correct identification does not interfere with tDCS results [57]. In addition, the present study only investigated the immediate effects of cathodal tDCS on clinical outcomes, and further investigation on the after-effects of tDCS is recommended. Moreover, the present study lacks physiological outcomes for the tDCS effects, and an investigation of physiological changes is suggested for the future.

## Conclusion

This study reported the non-linear effect of cathodal tDCS over the M1 on muscle strength in healthy participants. With intensities of 1 and 1.5 mA, tDCS resulted in the reduction of most of the upper and lower extremity muscle strength bilaterally. When the intensity was increased to 2 mA, the effect was reversed. The current results suggest a non-linear effect of cathodal tDCS as measured by motor outcome when the intensity is high (2 mA), which is consistent with previously reported neurophysiological changes [22, 26]. High-intensity tDCS is usually used to target deep cortical areas. These findings warrant an appropriate selection of cathodal tDCS intensity, especially at 2 mA or higher, in clinical practice, for example to rebalance IHI in stroke or to reduce involuntary movement in individual with neurological disorders.

## Supplementary Information

**Additional file 1: Table S1.** Adverse effects percentage of different cathodal tDCS intensities in healthy participants.

## Data Availability

All data generated or analyzed during this study are included in this published article.

## Abbreviations

IHI: Interhemispheric inhibition
IQR: Interquartile range
M1: Primary motor cortex
POST: Posttest
ROAST: Realistic volumetric approach to simulate transcranial electric stimulation
SD: Standard deviation
tDCS: Transcranial direct current stimulation
ctDCS: Cathodal transcranial direct current stimulation
