# Supplementary material for: Non-linear dose response effect of cathodal transcranial direct current stimulation on muscle strength in young healthy adults: a randomized controlled study
Source: BMC Sports Sci Med Rehabil. 2023 Jan 30;15:10. doi: 10.1186/s13102-023-00621-7 (PMC9887803; doi:10.1186/s13102-023-00621-7)
Supplement: Supplementary file 1 — Additional file 1: Table S1. Adverse effects percentage of different cathodal tDCS intensities in healthy participants. [file 13102_2023_621_MOESM1_ESM.docx]

**Additional file 1: Table S1** Adverse effects percentage of different cathodal tDCS intensities in healthy participants.

| **Adverse effects** | **1 mA ctDCS (%)** | **1.5 mA ctDCS (%)** | **2 mA ctDCS (%)** |
| --- | --- | --- | --- |
| Tingling | 66.67 | 58.33 | 58.33 |
| Skin redness | 16.67 | 33.33 | 75.00 |
| Sleepiness | 8.33 | 41.67 | 8.33 |
| Burning sensation | 8.33 | 8.33 | 25.00 |
| Itching | 0.00 | 8.33 | 25.00 |
| Headache | - | - | - |
| Neck pain | - | - | - |
| Scalp pain | - | - | - |
| Trouble concentrating | - | - | - |
| Acute mood change | - | - | - |
| Others | - | - | - |
